# “I Feel Closer to Her Now That I Know What She Went Through”: Findings from a Survey on Siblings’ Relationships Following Childhood Sexual Abuse

**DOI:** 10.1177/08862605251339635

**Published:** 2025-05-24

**Authors:** Rosaleen McElvaney, Simon Dunne, Laura Cahill, Rachael McDonnell Murray

**Affiliations:** 1 Dublin City University, Ireland; 2 The Alders Unit, Children’s Health Ireland at Connolly, Dublin, Ireland; 3 Dublin City University, Ireland

**Keywords:** sexual abuse, child abuse, family issues and mediators, child abuse, attachment, secondary/vicarious trauma

## Abstract

Limited attention has focused on sibling relationships following disclosure of childhood sexual abuse. This study sought to investigate changes, if any, in sibling relationships before, immediately following disclosure, and at the present time, from the perspective of non-abused adult siblings. A cross-sectional survey design was used to measure the extent of closeness, explore changes in relationships, and whether closeness varied according to other factors such as age, gender, birth order, or favoritism by parents. Analysis of 99 participant responses indicated that participants felt more close to their siblings following disclosure. Further analysis indicated that while participants felt more positive feelings toward their abused sibling when the abuser was intrafamilial, they felt less close to them than when the abuser was extrafamilial. The findings highlight the complexity of relational dynamics and emotional responses (e.g., ambivalence) in family relationships in general, and sibling relationships in particular, as family members manage the fallout of child sexual abuse. More research is needed to explore the impact of sexual abuse on this important family relationship.

Childhood physical and emotional abuse, particularly where the abuse was perpetrated by family members, has been found to have significant negative effects on the quality of family relationships across the lifespan (Schafer et al., 2011), specifically on closeness in family relationships (Savla et al., 2013). Conversely, positive family relationships, particularly mother–child relationships, have been recognized as having a buffering effect in terms of the psychological impact of sexual abuse (Zajac et al., 2015) and are associated with good outcomes for children engaging in therapy (Cohen & Mannarino, 2000). Less is known about the contribution of positive sibling relationships, yet as McHale et al. (2012) note, children in the United States are more likely to grow up in a family home with a sibling than with a father. Conte and Schuerman (1987) found that the lack of supportive relationships with siblings and non-offending parents was associated with parent-reported child psychopathology. Siblings have been largely overlooked in the research literature on child sexual abuse. Recent research on how siblings are impacted by sexual abuse, perpetrated by either someone within the family or outside, suggests that sibling relationships and the impact of sexual abuse on sibling relationships are fields that warrant further investigation (Crabtree et al., 2021; Stocker et al., 2020; Tanskanen & Danielsbacka, 2021). The current study sought to explore the perspectives of non-abused siblings on family relationships, what reported changes, if any, are experienced in these relationships with a view to improving our understanding of how families may need support following sexual abuse.

## Sibling Relationships

The sibling relationship has been described as the most enduring relationship across the lifespan (Cicirelli, 1995), reflecting ebbs and flows between closeness and distance, both at different stages of the life course, and in response to developmental tasks and life events. Siblings are often part of each other’s “inner circle” during childhood and “outer circle” during early adulthood, but may return to the “inner circle” in middle or late adulthood (Gilligan et al., 2020; Kong & Goldberg, 2022; Stocker et al., 2020). Their shared experiences in family life contribute to affectional bonds, trust, shared perceptions, and mutual understanding, which all influence attachment style (Ainsworth, 1989), in turn impacting how individuals experience other relationships in their lives across the lifespan (Dimitrova et al., 2009). However, sibling relationships vary according to many factors and have the potential to be experienced as a resource or a burden. Such factors include age difference and gender, personality traits, relationships with parents (such as favoritism), family environment and parental relationships, sibling roles, and social expectations (Witte et al., 2020). Conger and Little (2010) also referred to factors such as the number of children in a family, power and equality within the relationship, and cultural factors, such as expectations of older siblings or those of a particular gender, which have been much less studied.

Supportive, close, and warm sibling relationships have been found to buffer the impact of stressful life events on internalizing problems (Feinberg et al., 2012; Gass et al., 2007), independent of the quality of the mother–child relationship (Gass et al., 2007). In addition to sibling relationships representing a protective factor for mental health, these relationships have also been found to be impacted positively by adverse childhood experiences, by bringing siblings closer together, characterized by support and warmth in the relationship (Witte et al., 2020). However, Witte et al. (2020) also found that adverse childhood experiences could place more stress on the sibling relationship, characterized by conflict, and that child maltreatment increased the likelihood of a hostile and less harmonious sibling relationship pattern. Further exploration of how sibling relationships are experienced in these contexts could improve our understanding of both positive and negative impacts of adverse childhood experiences on sibling relationships.

## Sibling Relationships Following Sexual Abuse

Siblings have described blaming themselves for not being able to protect their sibling, both in child samples (Hill, 2003) and adult samples (Monahan, 1997). Baker et al. (2002) note that disclosure often brings stress on child siblings: how to respond to peers when asked questions; coping with negative reactions from neighbors or others in the community; coping with the stress of police investigations; disbelieving or resenting their sibling for disclosing the abuse; and needing to protect their sibling from further abuse. When abuse is perpetrated by a family member or frequent caregiver, non-abused siblings may live in fear of being abused themselves (Schreier et al., 2017). Where the perpetrator was a parent, rivalry, jealousy, and envy between abused and non-abused siblings have been reported, particularly in the context where increased attention, gifts or privileges were bestowed on the abused child (De Young, 1981; Monahan,1997).

In adulthood, siblings have been described as an important source of support for abused individuals (Katz & Tener, 2021). However, recent research conducted directly with adult non-abused siblings has revealed tensions in sibling relationships following the disclosure of childhood sexual abuse. Often such disclosures occur in adulthood and non-abused siblings have described divided loyalties when family members were the perpetrators of the sexual abuse (Monahan, 1997; Hill, 2003; Schreier et al., 2017; Crabtree et al., 2021; McElvaney et al., 2022 ). Adult non-abused siblings have reported experiencing distress as they are confronted with changed scripts of happy family life in childhood (Crabtree et al., 2021; McDonnell Murray et al., 2020; Welfare, 2008), while taking on significant support functions within their families. This has resulted in non-abused siblings feeling torn as they try to support family members—the victim, the offender and/or their parents—following the disclosure (Welfare, 2008; Schreier et al., 2017; Crabtree et al., 2021; McElvaney et al., 2022). Thus, siblings’ capacity to offer support may be compromised, depending on how they and other family members are impacted by the discovery of abuse, irrespective of whether the abuse occurred within the family or outside (Crabtree et al., 2021; Welfare, 2008).

Authors have called for support for siblings in the aftermath of disclosure, noting that the impact on parents has received more attention both in research and service provision. We know from research on intimate relationships that the capacity to preserve closeness and proximity in relationships represents a mediator between childhood sexual abuse and adult psychopathology (Dimitrova et al., 2009). It may be that relational closeness between siblings serves a similar function and can be a protective factor for those who have experienced childhood sexual abuse. To our knowledge, no studies to date have examined whether closeness in sibling relationships is itself impacted by the disclosure of CSA. Extant qualitative studies have indicated that for some, the discovery of abuse can change siblings’ perspectives of family relationships as the knowledge of the abuse forces them to reflect on these relationships, altering family scripts (Crabtree et al., 2021) and that sibling and other family relationships can suffer as a consequence (Crabtree et al., 2021; Welfare 2008). For instance, all five participants in Crabtree et al.’s (2021) phenomenological study described changes in both their sibling relationships and their relationship with their wider family following disclosure of sexual abuse. For some, the disclosure brought them closer, while for others, it led to tensions and deterioration in these relationships. Participants described communication and talking about the abuse as challenging, and while wanting to be supportive of their abused sibling, experienced difficulties in doing so. They described feeling silenced, which in itself motivated them to participate in the research study. The authors note that very limited attention has been paid to adult siblings and how the “long tail of abuse” (Crabtree et al., 2021) impacts their relationship with their abused sibling. Given the significant resource that siblings offer in the recovery journey (Katz & Tener, 2021), more needs to be understood about siblings’ perspectives on how their relationship has been impacted, from their perspectives. A better understanding of the impact of disclosure on closeness in sibling relationships could guide professionals in supporting siblings and those abused in negotiating family relationships following CSA disclosures.

The current study sought to examine more closely the sibling relationship from the perspective of the non-abused sibling, specifically on perceived changes, if any, experienced by them following the discovery of abuse experienced by their sibling; whether they experienced changes in relationships over time: before disclosure, following disclosure, and the present time; and whether their perception of closeness varied according to other factors such as age, gender, birth order, or favoritism by parents. Recent studies have explored in depth siblings’ experiences in small samples primarily accessed through support services; we sought to capture a wider representation of siblings from the general population to capture diversity in terms of age, gender, and family constellation to ascertain whether the findings from qualitative studies were illustrative of a larger population of non-abused siblings.

## Method

### Design

This study had a cross-sectional survey research design, with the intention of exploring the relationship between non-abused siblings and their abused siblings before and after a disclosure of abuse and in relation to a variety of characteristics of each sibling. The survey itself was divided into sections relating to: personal characteristics of the respondent, including demographic and family structure and composition questions (11 items); family relationships during childhood (9 items) and at the present time (9 items); details about the sexual abuse itself, including how the participant came to know about it and how family members responded to it (22 items); the alleged abuser’s response/reaction to the disclosure (7 items); aspects of the relationship to the abused sibling (36 items; described further below); and questions about the extent to which the sibling or participant engaged in counselling or psychotherapy (7 items). In several sections, additional text boxes allowed participants to input open-ended qualitative responses (specifically, open-ended text boxes accounting for relationship changes from childhood to present time, relationship changes specifically relating to the childhood sexual abuse, details of the sexual abuse, details of the alleged abuser’s response/reaction, and the type of contact maintained with the abused sibling). In the analysis that follows in the results section, information provided by participants in these latter questions is selectively used to contextualize statistical survey results.

For questions on the relationship to the abused sibling, in the absence of appropriate scales to investigate the closeness of sibling relationships, we constructed a series of items that attempted to capture various aspects of sibling relationships.^1^ Firstly, we adapted the single-item “Inclusion of Other in the Self (IOS) Scale” (Aron et al., 1992), which measures how close an individual feels with another person, in order to capture non-abused siblings’ general feelings of closeness to their abused sibling before the disclosure of abuse (one item) and at the present time (one item). For both items, the structure of answers was retained as per the original measure (7 response options depicted as “circles” of varying closeness). Secondly, we developed a series of items that attempted to represent four separate domains relating to non-abused siblings’ closeness to their abused sibling: level of contact with their sibling (4 items on a five-point Likert-type scale from “*at least once a week*” to “*less than once a year*,” e.g., “How often do you and this sibling spend time with each other?”; α = .93), the extent to which they provide emotional support to their sibling (13 items on a 7-point Likert-type scale from “*Strongly agree*” to “*Strongly disagree*,” e.g., “I try to cheer this sibling up when they are feeling down”; α = .89), the extent to which they provide supportive behaviors to their sibling at the current time (5 items on a 7-point Likert-type scale from “*Strongly agree*” to “*Strongly disagree*,” e.g., “I have offered suggestions to my sibling about how they can cope with the sexual abuse”; α = .61) and current feelings about their sibling (9 items on a 7-point Likert-type scale from “*Strongly agree*” to “*Strongly disagree*,” e.g., “I feel sad about my sibling being abused”); α = .81).

In constructing these questions, we drew on findings from qualitative research directly exploring siblings’ experiences (Crabtree et al., 2021), as well as a range of scales in order to adapt appropriate items relating to these domains. These included The Family Adaptability and Cohesion Scale IV (FACES IV; Olson, 2011), the Parental Support Questionnaire and the Parent Emotional Response Questionnaire (PERQ; Mannarino & Cohen, 1996), the Parental Reaction to Abuse Disclosure Scale (PRADS), and the Adult Sibling Relationship Questionnaire (ASRQ; Lanthier et al., 2000; Wallace, 2012). Where a particular domain was lacking in sufficient detail or content, we also created several bespoke items in line with Pett et al.’s (2003) principles of scale development (e.g., “I have told my sibling that I want their abuser punished for the sexual abuse”). The survey also included questions regarding demographic variables such as gender, age, family structures and relationships (original and present), as well as variables related to the abuse (type of abuse), disclosure details, and variables related to the sibling relationship (closeness, contact level, emotional and behavioral support, feelings about sibling). The race and ethnicity of participants were not collected, and given the growing diversity of ethnic backgrounds in the Irish population, this would have been helpful. Research does indicate the impact of family values and faith-based practices on sibling relationships across different ethnic and racial groups (Updegraff et al., 2011), which may be relevant for increasing our understanding of sibling relationships following sexual abuse.

### Procedure

Qualtrics was employed as an online platform for data collection. This allowed participants to anonymously complete the survey nationally, once they had access to the link. The link immediately directed potential participants to an information sheet, followed by an informed consent sheet. Those choosing not to participate were presented with a debriefing sheet, which included information about support services. Time to complete the survey varied depending on the depth of qualitative information provided, but on average varied between 20 and 30 min.

Participants for the larger study were recruited through a wide range of sampling strategies between 2019 and 2022. These strategies included contacting (a) key organizations in Ireland that provide services for people who have experienced CSA; (b) articles in 17 national and local newspapers (print and electronic) throughout Ireland; (c) speaking on eight national and local radio programs throughout Ireland; a Facebook page about the study and Facebook Sponsored Advertisements over a two-month period; (d) a recruitment poster on the first author’s website, with the accompanying link to the study; and (e) a recruitment poster in local libraries, General Practitioner and Dental practices in Dublin city. Participants were eligible to participate if they were over 18 years of age, had a sibling who experienced CSA, and were not a victim themselves. The study received institutional ethical approval from Dublin City University.

### Data Preparation and Analysis

Aggregated mean scores were created for individual participants’ current contact level with sibling, extent of emotional support, feelings about their sibling now, and the extent to which they provided supportive behaviors based on their responses to the Likert-type scale questions in relation to these constructs described above. Total frequencies, mean scores, and standard deviations were calculated for demographic variables. We compared closeness to the abused sibling before and after a participant discovered they were abused, using a repeated measures *t*-test. Due to the small numbers of participants and differences in group size, we compared participants on a number of sociodemographic and family-related characteristics using nonparametric statistical tests as appropriate (Mann–Whitney *U* or Kruskal–Wallis tests) in terms of variables related to the sibling relationship. For all tests, a *p* value less than .05 was considered statistically significant.

## Results

Sociodemographic characteristics of participants (*P*) and characteristics of the participants’ sexually abused sibling and wider family are provided in Table 1, and characteristics of the siblings’ sexual abuse and its disclosure are provided in Table 2. Of the 152 individuals who started the survey, 99 completed 75% of the questions. We therefore excluded data from 53 participants who completed less than 25% of the survey. The high attrition rate in this study is discussed later in this article. The descriptive data in the tables presented below relate to these 99 participants.

**Table 1.** Demographic Characteristics of Participants.

Variable	*N*	%
Gender		
Male	18	18.2
Female	80	80.8
Other	1	1.0
Age		
18–24	5	5.1
25–34	20	20.2
35–44	18	18.2
45–54	26	26.3
55–64	22	22.2
65+	2	2.0
Missing	6	6.1
Relationship status		
Single	19	19.2
Married/cohabiting	66	66.7
Separated/divorced	13	13.1
Widowed	1	1.0
Number of participant’s children		
None	19	19.2
1	15	15.2
2–3	41	41.4
4	10	10.1
Missing	14	14.1
Number of children in family of origin		
2–3	29	29.3
4–6	44	44.4
7+	26	26.3
Relationship to abused sibling		
Biological sibling	93	93.9
Twin	3	3.0
Adopted sibling	2	2.0
Missing	1	1.0
Birth order of participant in family of origin		
Eldest	21	21.2
Middle	55	55.6
Youngest	21	21.2
Missing	2	2.0
Birth order of abused sibling compared to participant		
Older	43	43.4
Younger	46	46.5
Twin	6	6.1
Missing	4	4.0
Any other sibling(s) abused as a child in family		
Yes	31	31.3
No	67	67.7
Missing	1	1.0
Gender of the other abused sibling		
Male	7	7.1
Female	8	8.1
Both male and female	6	6.1
More than one male	4	4.0
More than one female	5	5.1
Missing	69	69.7
Description of family structure		
Matriarchal	37	37.4
Patriarchal	25	25.3
Neither	36	36.4
Missing	1	1.0

**Table 2.** Characteristics of Sibling’s Sexual Abuse and its Disclosure (*n* = 99).

Variable	*N*	%
Type of abuse		
Noncontact abuse	1	1.0
Contact abuse	15	15.2
Attempted penetration/penetration	37	37.4
Does not know	10	10.1
Missing	36	36.4
When the participant discovered abuse		
Immediately after it happened	7	7.1
Within a year of it happening	8	8.1
Within a decade of it happening	14	14.1
More than a decade after it happened	34	34.3
Missing	36	36.4
Age when participant discovered abuse		
Under 12	7	7.1
12–16 years	10	10.1
17–24 years	17	17.2
25–34 years	14	14.1
35–64 years	16	16.2
Missing	35	35.4
Abused sibling’s age when participant discovered abuse		
Under 12	10	10.1
12–16 years	8	8.1
17–24 years	15	15.2
25–34 years	18	18.2
35–64 years	12	12.1
65+ years	1	1.0
Missing	35	35.4
How participant discovered abuse		
Participant witnessed the abuse	4	4.0
From abused sibling	36	36.4
From another sibling	5	5.1
From mother	10	10.1
Other	10	10.1
Missing	34	34.3
Relationship of sibling to abuser		
Nuclear family	20	20.2
Mother (*n* = 1, 5%)		
Father (*n* = 12, 60%)		
Brother (*n* = 17, 85%)		
Sister (*n* = 1, 5%)		
Extended family	6	6.1
Extrafamilial	13	13.1
Missing	60	60.6

### Inferential Analyses of Siblings’ Relationships

In the following sections, we present the results of the planned analyses laid out in the method. This comprises results relating to closeness before and after the abuse, and the relationship between participants on a number of sociodemographic and family-related characteristics (family size, birth order, gender, number of other siblings abused, participant relationship status, relationship to abuser, and disclosure time after the abuse) in relation to the aggregated scores on their current contact level with sibling, extent of emotional support, feelings about their sibling now, and the extent to which they provided supportive behaviors. Table 3 presents descriptive data (means and standard deviations) relating to participants’ relationship to their abused sibling by these characteristics. Where significant relationships between variables were identified, we purposefully explored the qualitative data to investigate potential explanations. Thus, in presenting the findings below, we include quotes to illustrate findings, where relevant.

**Table 3.** Descriptive Statistics Relating to Participants’ Relationship to their Abused Sibling by Sociodemographic, Relationship and Disclosure Characteristics.

Variable	Closeness Before *M* (*SD*)	Closeness Now *M* (*SD*)	Contact Level *M* (*SD*)	Extent of Emotional Support *M* (*SD*)	Feelings About Sibling Now *M* (*SD*)	Extent of Supportive Behaviors *M* (*SD*)
Total	3.18 (1.39)	3.61 (1.80)	2.74 (1.49)	4.34 (1.58)	5.25 (1.04)	5.39 (1.28)
Family size						
2–3 children	3.49 (1.52)	3.63 (1.88)	2.83 (1.51)	4.41 (1.60)	5.00 (0.78)*	5.34 (1.58)
4+ children	3. (1.62)	3.11 (1.83)	2.10 (1.51)	3.36 (2.05)	5.94 (1.02)*	5.11 (1.55)
Birth order of sibling						
Older	3.05 (1.47)	3.35 (1.66)	2.34 (1.77)	3.73 (2.29)	5.32 (1.18)	5.24 (1.37)
Younger	3.53 (1.30)	3.38 (1.72)	3.09 (1.42)	4.50 (0.92)	4.88 (0.94)	5.45 (1.21)
Twin	4.33 (1.50)	3.00 (1.41)	4.75	5.08	5.4	6.62
Gender of participant						
Male	3.29 (1.27)	3.67 (1.60)	2.48 (1.5)	4.52 (1.69)	5.02 (1.29)	5.34 (1.30)
Female	3.41 (1.44)	3.37 (1.64)	2.84 (1.48)	4.30 (1.58)	5.30 (0.99)	5.40 (1.30)
Gender of abused sibling(s)						
Male	3.50 (1.31)	3.20 (1.69)	2.58 (1.45)	3.93 (1.70)	5.47 (0.91)	5.32 (1.46)
Female	2.64 (1.39)	3.11 (1.62)	2.31 (1.51)	4.18 (2.01)	5.80 (1.30)	5.28 (1.11)
Other siblings abused						
Yes	3.00 (1.41)	2.90 (1.37)	2.07 (1.54)	3.93 (1.71)	5.00 (0.94)	4.77 (1.02)
No	3.52 (1.39)	3.57 (1.73)	3.02 (1.62)	4.18 (2.01)	5.13 (1.11)	5.59 (1.25)
Relationship status (participant)						
Single	3.50 (1.10)	3.28 (1.64)	3.06 (1.39)	4.92 (0.95)	4.85 (0.94)	5.63 (0.93)
Married/cohabiting	3.42 (1.45)	3.56 (1.57)	2.62 (1.51)	4.15 (1.77)	5.35 (1.04)	5.35 (1.36)
Divorced/separated	2.80 (1.48)	2.71 (2.06)	2.75 (1.78)	4.23 (0.80)	5.20 (1.31)	4.92 (1.46)
Widowed	5.00	1.00	3.75	5.30	5.00	6.50
Relationship to abuser						
Nuclear family	3.50 (1.21)	3.80 (1.74)*	2.11 (1.52)**	3.83 (1.78)**	5.71 (0.94)*	5.21 (1.20)
Extended family	3.50 (2.07)	1.80 (1.30)*	3.20 (0.95)**	4.71 (0.86)**	5.07 (1.04)*	5.45 (1.39)
Extrafamilial	3.50 (1.51)	4.00 (1.41)*	3.42 (1.40)**	4.85 (1.39)**	4.17 (0.80)*	5.63 (1.40)
Disclosure time after the abuse						
Immediately after	3.86 (1.57)	5.50 (1.00)	3.00	5.85	3.40	5.87
Within a year	3.63 (0.92)	2.63 (1.77)	4.13 (0.88)	4.85 (0.18)	4.20 (0.80)	5.09 (0.57)
Within a decade	4.07 (1.27)	3.77 (1.53)	2.33 (2.15)	3.13 (3.04)	5.73 (0.99)	5.62 (1.21)
Over a decade	2.92 (1.30)	3.37 (1.50)	2.75 (1.62)	4.01 (1.53)	5.57 (1.06)	5.06 (1.20)

* *p* < .05. **p<.01.

#### Closeness Before and After the Abuse

A repeated measures *t*-test revealed a just significant effect [*t*(67) = −1.993, *p* = .05; Cohen’s *d* = 0.06], indicating that siblings were closer with their abused sibling after they found out about the abuse (*M* = 3.60; *SD* = 1.81), compared to beforehand (*M* = 3.19, *SD* = 1.27). There was some indication from the qualitative data that the disclosure in itself brought closeness to the relationship, insofar as open communication helped with closeness, “I feel closer to her now that I know what she went through. My whole understanding of her childhood, and mine, was dramatically altered when I found out what happened to her when we were kids.” (P134, 2–3 children in family of origin (FOO), abuser from nuclear family); “her being able to talk about it more has bonded us closer.” (P98, 4–6 children in FOO, abuser was extrafamilial); “we are able to openly discuss the fallout and longer term impact on our family, our emotions (P77, 7+ children in FOO, abuser from nuclear family). Poor communication, conversely, was described as contributing to the lack of closeness for some individuals, “She has distanced herself from family, does not speak openly about it to us, does not attend family occasions, is angry about our response to her disclosure . . . she does not feel supported and believed” (P5, 2–3 children in FOO, abuser from nuclear family). Thus, the ability on the part of the abused sibling to talk about the experience and the impact of the abuse may bring siblings closer together.

#### Comparing Closeness and Related Variables by Relationship of Sibling to Abuser

We investigated whether siblings’ relationship to the abuser (nuclear family member vs. extended family member vs. extrafamilial) was associated with participants’ perception of closeness toward their sibling before the abuse or the extent to which they provide supportive behaviors toward their abused sibling. Kruskal–Wallis tests revealed no significant differences. However, a statistically significant difference was found in closeness to the abused sibling at the present time (Gp1, *n* = 26: nuclear family; Gp2, *n* = 12: extended family; Gp3, *n* = 18: extrafamilial), χ^2^ (2, *n* = 56) = 7.35, *p* < .05). The extrafamilial group recorded a higher median score (*Md* = 5) than the other two groups (nuclear family, *Md* = 3; extended family, *Md* = 3.5). Post hoc Mann–Whitney *U* tests revealed no significant differences between extended family members and abusers who were extrafamilial on current closeness to their sibling (*p* > .05) but did reveal a significant difference between nuclear family members and abusers who were extrafamilial on this variable; *U* = 123, *z* = –2.71, *p* < .05, *r* = .41. Thus, participants reported feeling closer to their abused sibling at the present time when the abuser was extrafamilial than when the abuser was a member of the nuclear family. It appears that in some cases, the fallout from the discovery of the abuse brought families closer together, “Aunts and uncles chose their side and we do not speak to most of them anymore. But that just meant that my immediate family are closer than ever” (P98, 4–6 children in FOO, abuser was extrafamilial).

The finding that participants felt less close to their abused sibling when the abuser was a member of their nuclear family may be related to the perceived challenge of supporting the abused sibling, “ you cannot fix it for them, and you have to mind yourself too” (P117, 3 siblings, extended family); a sense that the abused sibling is getting more support than they are, “If abuse occurs within a family we must be aware that an abuser grooms a family, not just their victim. Do not blame your (abused) sibling if they are suddenly getting far more emotional support than you are, especially if you are young and still reliant on your family. Do not. Blame.” (P30, 2–3 children in FOO, abuser within extended family); or guilt that the participant was not abused themselves, “I feel guilty that I was not a victim myself.” (P34, 8 children from FOO, abuser from nuclear family). It may also be related to differences in feelings toward the abuser. One participant, whose sibling was abused by their father, captured this conflict, “I spoke with her and said that he (father) had destroyed her life. She responded that he had not. It is strange that for many years my sister and her partner would often go on 2-week holiday abroad with my father and mother. . .I never understood it!” (P98, 5 children within FOO, abuser within nuclear family).

The impact of the discovery of the abuse on non-abused siblings may have impacted on closeness in the sibling relationship, such as having to keep the secret, “I feel like I am carrying a BIG shameful secret with me every day and I hold back from getting too close to people in case I would ever say something to them” (P104, 9 siblings in FOO, abuser from nuclear family). Finally, the impact of the CSA on the abused sibling may also have placed a strain on sibling relationships, “We were estranged for approx 5 years because I doubted her. . .She has a history of erratic behavior that I realized was not simply manipulating and lying but was clinical due to her diagnosis that was caused by her abuse. It is still a difficult road to recovery in our relationship (P151, 2–3 children in FOO, abuser from nuclear family).

A Kruskal–Wallis Test revealed a statistically significant difference in the level of current contact with the abused sibling (Gp1, *n* = 29: nuclear family; Gp2, *n* = 12: extended family; Gp3, *n* = 17: extrafamilial), χ^2^ (2, *n* = 58) = 10.16, *p* = .006). The extrafamilial group recorded a higher median score (*Md* = 4) than the other two groups (nuclear family, *Md* = 1.75; extended family, *Md* = 3.25). Post hoc Mann–Whitney *U* tests revealed no significant differences between extended family members and abusers who were either extrafamilial or nuclear family members (*p* > .05) on the level of current contact with the abused sibling but did reveal a significant difference between nuclear family members and abusers who were extrafamilial on this variable; *U* = 121, *z* = –2.87, *p* < .05, *r* = .42. Thus, participants reported that they had more contact with the abused sibling at the present time where the abuser was from outside the family, than if the abuser was a member of the nuclear family. One participant spoke of struggling with their own mental health and that they have “no contact with either sibling anymore. [I also have] no contact with their children (my nephews)” (P136, no family size provided, abuser from nuclear family).

A Kruskal–Wallis Test revealed a just statistically significant difference in the extent of emotional support provided to the sibling at the current time (Gp1, *n* = 29: nuclear family; Gp2, *n* = 12: extended family; Gp3, *n* = 17: extrafamilial), χ^2^ (2, *n* = 58) = 5.992, *p* = .05. The extrafamilial group recorded a higher median score (*Md* = 5.08) than the other two groups (nuclear family, *Md* = 4.15; extended family, *Md* = 4.69). Post hoc Mann–Whitney *U* tests revealed no significant differences between extended family members and abusers who were extrafamilial on the extent of emotional support they presently provide to their sibling (*p* > .05) but did reveal a significant difference between nuclear family members and abusers who were extrafamilial on this variable; *U* = 147.5, *z* = –2.26, *p* < .05, *r* = .33. One participant reported providing a lot of emotional support to their sibling when the abuser was from outside the family,

As a child I didn’t fully understand the abuse. I don’t know the full extent, but I do know it was rape and oral sex involved. {. . .} my brother was only 4. Now, as an adult, I feel protective of my sibling and wish I could change the past. (P96, 4–6 children in FOO, abuser was extrafamilial)

while another, whose sibling was abused by a nuclear family member, spoke of tension in the relationship with their abused sibling, and avoidance of discussing certain topics relating to their childhood “My sibling can be very cutting/negative about other family members and about childhood memories which I remember as happy memories so I avoid discussing those things” (P52, 2–3 children in FOO, abuser from nuclear family).

Finally, a Kruskal–Wallis Test revealed a statistically significant difference in current feelings toward the abused sibling (Gp1, *n* = 29: nuclear family; Gp2, *n* = 12: extended family; Gp3, *n* = 17: extrafamilial), χ2 (2, *n* = 58) = 10.13, *p* = .006. The nuclear family group recorded a higher median score (*Md* = 5.8) than the other two groups (nuclear family, *Md* = 4.6; extended family, *Md* = 4.6). Post hoc Mann–Whitney U tests revealed no significant differences between extended family members and nuclear family members (*p* > .05) on current feelings of closeness toward sibling but did reveal a significant difference between nuclear family members and abusers who were extrafamilial on this variable, indicating participants had more positive feelings toward their abused sibling when the abuser was a member of the nuclear family than if they were from outside the family (*U* = 115, *z* = –3.05, *p* < .05, *r* = .45).

#### Comparing Closeness and Related Variables by Family Structures and Relationships

Comparisons between participants from small (1–3) and large (4 or more) families in relation to their feelings of closeness toward the sibling before the abuse, their feelings of closeness toward the sibling after the abuse, their current levels of contact with the sibling, the extent to which they provide emotional support to the participant, and the extent to which they provide supportive behaviors toward their sibling revealed no significant differences. However, in relation to current feelings about the abused sibling, a Mann–Whitney *U* test revealed a significant difference, indicating that participants from larger families (*Md* = 6.2, *n* =10) exhibited more positive feelings toward their siblings than participants from smaller families (*Md* = 4.6, *n* = 23), *U* = 57.5, *z* = –2.35, *p* < .05, *r* = .41. Qualitative data did not reference the size of families or how this may have impacted respondents’ feelings of closeness toward their siblings.

After removing two participants whose abused sibling was their twin, Mann–Whitney *U* tests revealed no significant differences in birth order (younger vs. older siblings), gender, number of siblings abused (one sibling abused vs. more than one sibling abused) and relationship status of the participant (in a relationship or not) in relation to participants’ feelings of closeness toward the sibling before the abuse, their feelings of closeness toward the sibling after the abuse, their current levels of contact with the sibling, the extent to which they provide emotional support to the participant, current feelings about the abused sibling, and the extent to which they provide supportive behaviors toward their sibling.

#### Comparing closeness and related variables by disclosure time after the abuse

Kruskal–Wallis tests revealed no significant differences in the period of time when the participant found out about their sibling’s abuse (immediately afterward vs. within a year vs. within a decade vs. over a decade) in relation to participants’ feelings of closeness toward the sibling before the abuse, their feelings of closeness toward the sibling after the abuse, their current levels of contact with the sibling, the extent to which they provide emotional support to the participant, current feelings about the abused sibling, and the extent to which they provide supportive behaviors toward their sibling.

## Discussion

This study sought to explore, through survey methods, closeness in sibling relationships and how this may or may not have changed, from the perspective of non-abused siblings, following the discovery of sexual abuse. Participants indicated that they felt they were closer to their abused siblings after they found out about the abuse compared to beforehand. They reported that they had more contact with and provided more emotional support to their abused sibling at the present time when the abuser was extrafamilial than when the abuser was a member of the nuclear family, but had more positive feelings toward their abused sibling when the abuser was a member of the nuclear family than if they were from outside the family. We also found that participants from larger families exhibited more positive current feelings toward their siblings than participants from smaller families. The other variables that we examined (including gender of either sibling, time between abuse and disclosure, birth order of the siblings, and number of abused siblings in the family) did not have an impact on any of our measures of perceived sibling closeness.

The findings reveal some interesting family dynamics that enlighten our understanding of sibling relationships following sexual abuse. Our expectation that there would be reports of changes in closeness in sibling relationships before and after the abuse was discovered was upheld; participants described feeling significantly closer to their abused sibling after the discovery of abuse than before. The qualitative responses to open questions about perceived changes in relationships referred to the participant understanding their sibling better, feeling protective toward them, and improved communication between them and their sibling following the disclosure, from their perspective. The disclosure itself may have contributed to the increase in closeness. Sharing confidences has been found to positively impact closeness in sibling relationships in adulthood (Hamwey et al., 2019; Myers, 2011). The increased contact and emotional support reported by those whose sibling was abused by someone outside the family may also have been associated with a feeling of protectiveness toward the abused sibling, as some participants referred to their sibling’s mental health difficulties and conflicts within the wider family, although those comments were also noted when the abuse was intrafamilial. Given the age profile of participants (50% of participants were over 45 years of age), this feeling of protectiveness may be related to the participant’s role in the family of supporting their adult siblings in the context of aging parents (McGoldrick et al., 2010).

The finding that there was more contact and more emotional support but less feelings of closeness when the abuser was a family member suggests that closeness did not necessarily correspond with increased contact with and emotional support for the abused sibling. A number of factors may explain this apparent contradictory pattern of findings. Firstly, as noted above, it may be that the vulnerability of the abused sibling elicited a response of protectiveness and regular contact but not necessarily closeness if the experience of supporting the sibling was experienced as challenging. For example, one participant in the study spoke of avoiding talking about their childhood with their abused sibling so that their own memories would not be contaminated. Crabtree et al. (2021) also found that non-abused siblings questioned their previously treasured childhood memories, which were now tainted with the knowledge of the abuse. These authors highlighted the challenges of supporting their abused sibling, sometimes due to their mental health difficulties, sometimes due to the psychological impact of the disclosure on themselves, and sometimes due to strained relationships in the family following the disclosure, particularly where abuse had occurred within the family. Similar themes were evident in the qualitative data in the current study. Secondly, it may be that non-abused siblings carry an additional burden in terms of family obligations that is not shared equitably with abused siblings. Participants in the current study reported feeling torn between wanting to support their sibling and wanting to maintain equilibrium in family relationships. Parker et al. (2018) found that a history of childhood abuse negatively impacted adults’ sense of obligation toward their families; severe physical and emotional abuse in childhood predicted lower levels of obligations compared with those who had no history of childhood abuse. It may be that a diminished sense of family obligation on the part of the abused sibling impacted sibling relationships, although there was no direct reference to this in the data from this study.

Thirdly, the apparent contradiction of more contact and emotional support but less feelings of closeness may reflect an experience of ambivalence, feeling conflicting emotions toward the abused sibling, both wanting to protect them and at the same time feeling angry toward them (Crabtree et al., 2021). According to Greif and Woolley (2016), ambivalence is inherent in adult sibling relationships, impacted by childhood rivalries, parental favoritism, and family interactions as well as personality differences, which change across the lifespan. It may be that this ambivalence is exacerbated in the context of sibling sexual abuse (SSA); most of the intrafamilial abuse described in this study related to abuse by a sibling (85%). Research exploring parent responses to sibling sexual abuse has highlighted considerable ambivalence in emotional responses to CSA within the family, impacting parent–child relationships, not only toward the adolescent who engages in sexually harmful behavior (Hackett et al., 2014; Tener et al., 2021), but also toward the child who discloses this within the family unit (Archer et al., 2020; McCoy et al., 2022; Tener et al., 2018). The limited research on the experiences of non-abused siblings suggests this is an area worthy of further investigation.

Respondents from larger families reported significantly more positive current feelings toward their siblings than smaller families. However, these results should be interpreted with caution, given the nonsignificant but higher mean results among smaller families across the closeness, contact level, and behavioral and emotional support variables. Few studies have examined family size as a variable influencing closeness in sibling relationships. While more positive attitudes and emotional closeness have been found to feature more in sibling relationships in larger families than in smaller families (Riggio, 2006), a recent review of literature on adult sibling relationships, though noting the mutual influence of various family structural variables such as gender and biological relatedness (Gilligan et al., 2020), made no reference to family size. Further research is needed to see if the results from the current study hold in a larger sample of siblings and, if so, to clarify why siblings from large families may experience more positive emotions toward their abused sibling than those from smaller families.

How closeness is defined is worth consideration. We examined related variables such as feelings of closeness to their sibling, positive feelings toward their sibling, levels of contact, provision of emotional support, and supportive behaviors toward their sibling. Frequency of contact, emotional support, and provision of help are often used as indicators of the quality of sibling relationships (Tanskanen & Danielsbacka, 2021). However, feelings of closeness did not necessarily correspond in this study with positive feelings toward siblings or an increased level of contact in our study. Of interest, Hamwey et al. (2019) found that diminished frequency of communication and time spent with siblings was associated with increased feelings of intimacy with a sibling. It may be that the concept of closeness in sibling relationships and what this means to siblings is worthy of further exploration.

### Strengths and Limitations

To our knowledge, this is the first study of its kind to recruit a sample of siblings from the general population through an anonymous survey gather information about disclosure and family relationships, and examine in depth on closeness in relationships from non-abused siblings’ perspectives.

While we made significant efforts to try to achieve a substantial sample that could capture the diversity in Irish society in terms of age, gender, and family constellation, only 152 individuals started the survey, and of these individuals, only 57 completed the full survey, although 99 completed over 75% of the questions. This sample size was smaller than anticipated, given the extensive methods used to recruit participants, and prohibited a comparison of different age cohorts and planned parametric inferential statistical analysis. The small sample is, in itself, a noteworthy finding, given the prevalence of childhood sexual abuse in Ireland (20%; Central Statistics Office, 2022), larger family size in Ireland compared to many other European countries (Eurostat, 2021; Testa, 2012), and extensive efforts to recruit participants from the general population.

Of note, a substantial proportion (25%) of those who began the survey discontinued, the largest cohort of dropouts being those who completed the first few demographic questions but did not continue to respond to questions about their own gender or who was favored within the family. Although the remaining participants did not answer all questions, they did continue the survey to the end. While the information sheet clearly outlined the purpose of the questionnaire, it may be that the act of completing the survey was too uncomfortable to sustain participation. Participants in Crabtree et al.’s (2021) study noted that their motivation to participate was influenced by their experience of feeling silenced. However, it may be that others feel torn between wanting to participate (through responding to the invitation) and sustaining their participation (through dropping out before reading the questions directly focused on the impact of the abuse). Murdoch et al. (2017) suggest that survey recipients who opt out of responding to surveys are engaging in self-protective behaviors. Trauma symptoms can be re-triggered at any time in an individual’s life, including during research (Batten & Naifeh, 2012). However, a meta-analysis by Jaffe et al. (2015), which reviewed data from 73,959 participants from 70 samples, concluded that while participants with PTSD were more likely to experience increased distress participating in research, overall, individuals do not feel retraumatized as a result of their involvement in trauma research.

The current study is also limited in its focus on the non-abused sibling’s perspective alone. It is important to consider the perspectives of both non-abused siblings and their abused sibling when exploring dynamics such as sibling relationships. Research with sibling dyads could elaborate on the findings from the current study and explore ambivalence within the adult sibling relationship and how CSA may impact those dynamics.

Cultural or religious diversity was not explored in this study. Western cultures tend to be more individualistic, with family units being self-contained. This can differ from Eastern cultures, which tend to have a more collectivist way of being, with intergenerational family living being commonplace (Davis & Williamson, 2020). Research does show that this influences the family unit, and specifically closeness in sibling relationships, as highlighted in Holmes et al.’s (2024) research into Latino family units. Further research would be helpful to build on findings collected here to understand the influence of race, ethnicity, and religion on the quality of sibling relationships, their closeness, following disclosure of child sexual abuse. Finally, the findings presented here are also limited by their cross-sectional nature and involve retrospective accounts (including changes in relationship closeness), which may be subject to inaccuracies in recall, including biases relating to memory and the impact of disclosure. As Gilligan et al. (2020) note, sibling relationships vary across the lifespan; longitudinal studies that follow sibling relationships from early adulthood through to later adulthood could shed light on the dynamic nature of this relationship.

## Conclusion

Positive sibling relationships are recognized as a protective factor for both abused and non-abused siblings across the lifespan. While participants in this study described feeling closer to their abused sibling following disclosure, this was significantly more of a feature where the abuser was someone outside the nuclear family than where the abuser was a family member. The impact of intrafamilial abuse on family relationships in general, and sibling relationships in particular, requires further exploration to inform understandings of the nuances of sibling relationships and the impact of sexual abuse, both intrafamilial and extrafamilial, on these important familial relationships over the lifespan. Assumptions may be made about sibling relationships based on expressed positive feelings toward, regular contact with, and emotionally supportive behavior toward abused siblings; however, non-abused siblings’ own feelings about the abuse and the family’s response to the abuse need to be taken into account in understanding what motivates these attitudes and behaviors toward abused siblings. The impact of abuse, particularly intrafamilial abuse, on non-abused siblings themselves and their experiences and perceptions of family relationships requires further exploration to better understand the role of non-abused siblings as family members age across the lifespan and how non-abused siblings can be supported. Services for adult survivors typically focus on the abused individual themselves, with limited services available to support the wider family. A greater appreciation of how both abused and non-abused individuals can be supported in nurturing their sibling relationship, and other family relationships, particularly when the abuser is a member of the nuclear family, could assist with maximizing the relationship for mutual benefit and healing for all family members. Further research exploring the meaning of closeness in sibling relationships, including perspectives of both abused and non-abused siblings and exploring experiences of both intrafamilial and extrafamilial abuse, could add to our understanding of this important relationship and how it can mediate the long-term impact of childhood sexual abuse.

## Footnotes

1 The full survey is available upon request.
